# Management of malignant bowel obstruction in patients with advanced cancer at the end of life

**DOI:** 10.2478/raon-2026-0010

**Published:** 2026-03-04

**Authors:** Nena Golob, Rok Petric, Maja Ebert Moltara

**Affiliations:** 1 Department of Acute Palliative Care, Institute of Oncology Ljubljana, Ljubljana, Slovenia; 2 Faculty of Medicine, University of Ljubljana, Ljubljana, Slovenia; 3 Divison of Surgery, Institute of Oncology Ljubljana, Ljubljana, Slovenia

**Keywords:** malignant bowel obstruction, advanced cancer, end of life, treatment, quality of life

## Abstract

**Background:**

Malignant bowel obstruction in patients with advanced cancer at the end of life is common. Patients may have substantial symptoms (pain, nausea and vomiting) and experience aggressive care at the end of life. Due to the lack of robust evidence, the care algorithm of treatment for patients at the end of life is not standardized. Patient’s prognosis, clinical condition and patient’s preferences related to goals of care discussion must be considered when opting between comfort-focused care (conservative/pharmacological treatment), palliative (nonsurgical) procedures and palliative surgery.

**Patients and methods:**

A focused literature search was conducted in PubMed/Medline to identify recommendations on the management of malignant bowel obstruction in patients with advanced cancer at the end of life. The search focused on symptom management using Medical Subject Headings (MeSH) terms related to intestinal obstruction in patients with advanced cancer at the end of life.

**Results:**

Using the MeSH terms related search in PubMed/Medline, 9,532 articles were initially identified. After applying filters, 535 articles were selected for further review. Additional sources included reference lists and grey literature. In total, 83 references were used to support the management recommendations/suggestions in this article.

**Conclusions:**

It is essential to engage patients and families in goals of care discussions to promote understanding of the palliative intent of different malignant bowel obstruction interventions. Research is needed to assist clinicians in decision making to provide patients at the end of life with appropriate care. Criteria for the selection of candidates for palliative surgery are needed to avoid significant complications and overly aggressive treatment at the end of life when the focus is maintaining and enhancing the quality of life of patients.

## Introduction

Malignant bowel obstruction (MBO) in patients with advanced cancer is common.^1–3^ Tumour seeding in the abdominal cavity, leading to peritoneal carcinomatosis, is a frequent pathway of cancer metastasis.^4,5^ Up to 25% of patients with advanced ovarian and colorectal cancer develop an intestinal obstruction in the course of their disease.^1–3,6–8^ Typically, it occurs at the end of life (EoL) with a survival of 1–2 months in patients with an inoperable disease and 3–8 months in surgically managed patients.^9^ Patients may have substantial symptoms and often receive aggressive care at the EoL.^4^ The care algorithm, especially for patients at the EoL, is not standardized and often varies based on individual cases with respect to primary cancer site, level of obstruction, comorbidities, patient functional status, and goals of care.^10^ The available literature guiding therapy for MBO is limited. Most publications are retrospective studies conducted at single tertiary centres, often reflecting the experiences of a highly selected population over extended periods. The prospective studies are typically uncontrolled observational studies focused on a single therapeutic approach. Due to the lack of robust evidencebased guidelines, physicians must often rely on personal experience to guide treatment decisions – resulting in significant variability in management strategies and patient outcomes.^11^ Although evidence remains limited for patients approaching the end of life, the Multinational Association for Supportive Care in Cancer (MASCC) multidisciplinary evidence-based recommendations/suggestions for the management of MBO offer structured guidance that can support clinical decision-making. These guidelines emphasize individualized, goal-concordant care, which is particularly relevant when balancing symptom relief, quality of life (QoL), and the burden of invasive interventions in EoL settings.^10^ Thus, the goals of care discussions are critical to align interventions – ranging from comfort-focused care (conservative/pharmacological treatment) to palliative surgery – with patient preferences and feasible options in mind.^12^ While several studies show benefit from palliative surgery with stoma/bypass/resection for relieving symptoms of obstruction, these interventions of unclear durability are many times related to high complication rates and morbidity.^9,13,14^ Recurrent and refractory obstruction is not uncommon and results in additional symptom burden for patients at the EoL where the focus of care is the QoL.^15^ Selfexpanding metallic stents were introduced in the 1990’s as an alternative to palliative surgery in palliative patients although peritoneal carcinomatosis (multiple MBOs) may represent a relative contraindication.^16,18^ Conservative therapy tends to have lower morbidity but does not extend survival significantly.^12^ Caring for patients with advanced cancer at the EoL and MBO thus requires a thoughtful, individualized approach tailored to each patient’s unique needs and circumstances.^19^

This review aims to underscore the importance of a multidisciplinary personalized approach and care in managing MBO in patients with advanced cancer at the EoL, with a primary focus on enhancing the QoL.

## Patients and methods

To identify recommendations regarding the management of MBO in patients with advanced cancer at EoL a focused literature research was performed in PubMed/Medline database, from inception to 29 July 2025, applying the following key words: Intestinal obstruction [Medical Subject Headings (MeSH)] OR Intestinal obstruction [title and abstract citation, tiab] AND (Pain [MeSH] OR Pain Management [MeSH] OR Nausea [MeSH] OR Vomiting [MeSH] OR Pain [tiab] OR Pain Management [tiab] OR Nausea [tiab] OR Vomiting [tiab]. Imposed restrictions for PubMed/Medline were as follows: abstract, book and documents, meta-analysis, practical guideline, randomized control trial, systemic review, English, humans and adult. The initial search strategy was broad, articles identified through this search were then manually screened to include only those addressing MBO in patients with advanced cancer – particularly those at the EoL – because our focus was the management of MBO-related symptoms, specifically pain, nausea, and vomiting, in this patient population.

## Results

Applying the following key words: Intestinal obstruction [Mesh] OR Intestinal obstruction [tiab] AND (Pain [Mesh] OR Pain Management [Mesh] OR Nausea [Mesh] OR Vomiting [Mesh] OR Pain [tiab] OR Pain Management [tiab] OR Nausea [tiab] OR Vomiting [tiab]) in PubMed/Medline database search, 9,532 articles were identified. Using the imposed restrictions (abstract, book and documents, clinical trial, meta-analysis, randomized control trial, review, systemic review, English, humans and adult: 19+), 535 articles were selected for further review. Only articles with an available abstract and in English language were reviewed. The articles were identified as relevant when addressing symptom management – pain, nausea and vomiting – in MBO in patients with advanced cancer at the EoL. Based on this relevance screening, 513 articles were excluded, leaving 22 articles that met the inclusion criteria.

**FIGURE 1.**
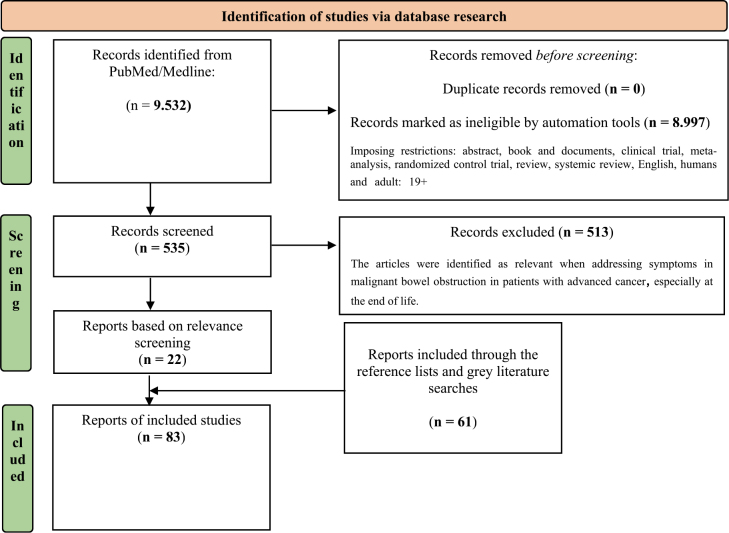
PRISMA flow diagram of study selection.

To ensure completeness, additional sources were identified through the reference lists of these articles and through grey literature searches. After integrating these supplementary materials, a total of 83 references were ultimately included to support the management recommendations presented in the article. The final reference set therefore consists of the 22 eligible articles plus additional relevant sources identified through reference-list review and grey-literature searching (Figure 1).

## Discussion

It is challenging to choose between comfort-focused care (conservative/pharmacological treatment), palliative (non-surgical) procedures and palliative surgery for physical symptoms relief as current evidence does not provide any gold standard of care in patients with advanced cancer at the EoL.^7,10^ The MASCC emphasized the need for a multidisciplinary holistic approach to manage MBO in patients with advanced cancer.^10^

**FIGURE 2.**
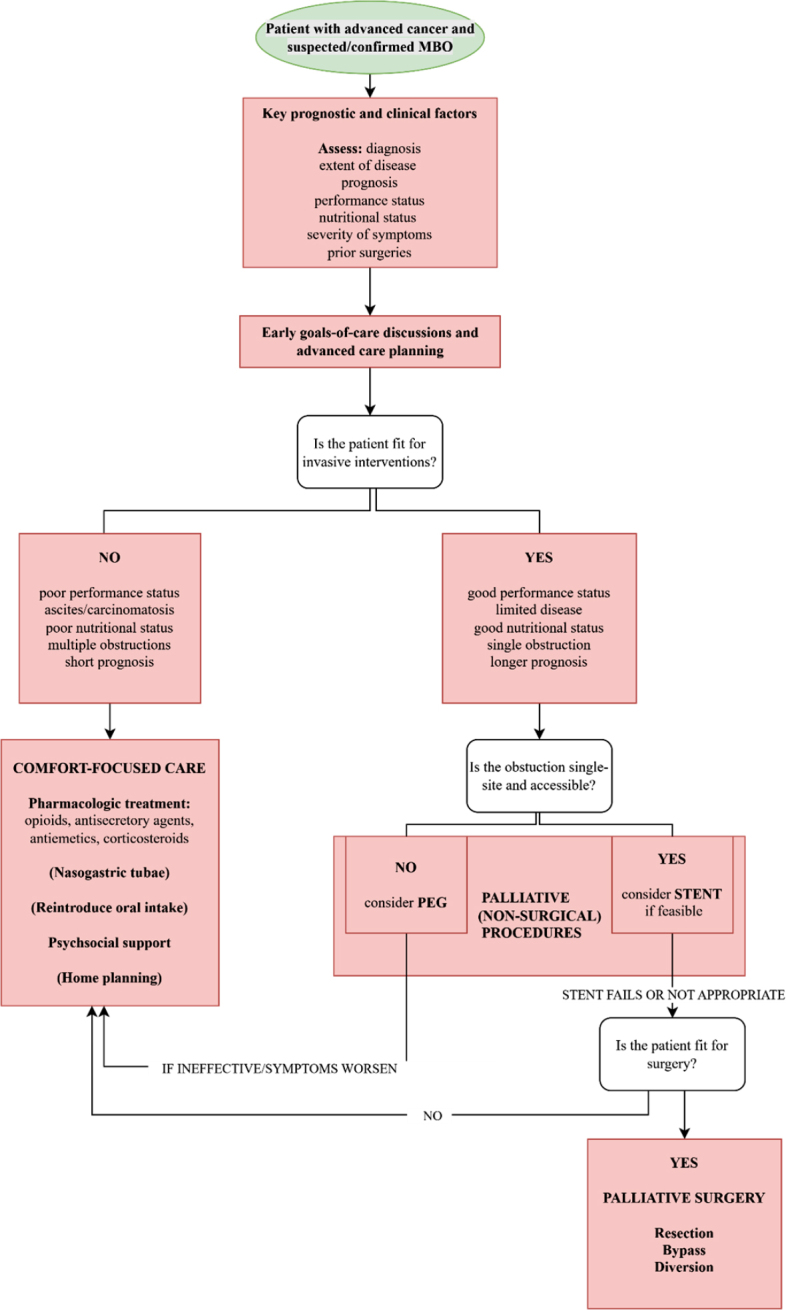
Therapeutic alghoritm of malignant bowel obstruction. MBO = malignant bowel obstruction; PEG = percutaneous endoscopic gastrostomy

Most studies available and the studies used in this article on MBO in advanced cancer rely on heterogeneous patient populations, making it difficult to account for the distinct needs and trajectories of individuals nearing the EoL. In many cases, specific EoL subgroups – in general and in our article – are not clearly differentiated within these analyses. As a result, clinical recommendations for patients who are unequivocally at the EoL stage are often extrapolated from broader and more diverse study populations, rather than based on evidence tailored directly to this group. Recognizing this gap, the algorithm proposed aims to offer a structured, EoL-focused approach to MBO management, supporting more goal-concordant, context-specific decision-making for this distinct patient group (Figure 2).

Bowel obstruction in advanced cancer is usually mechanical and may be partial or complete at single or multiple locations with small bowel more commonly involved (affected in 2/3 of cases) compared to large bowel.^2,3,20^ MBO may be the first presentation of advanced cancer.^8^ It normally develops gradually, though symptoms may appear suddenly.^19^ The main symptoms of bowel obstruction are abdominal pain, colic, nausea and vomiting.^3,21^ Distressing symptoms usually necessitate complex medical intervention.^3^

In the diagnostic process of MBO (X-rays), the contrast agent diatrizoate meglumine was proposed as a diagnostic agent with potential therapeutic effect possessing high osmotic activities and mild laxative effect.^22^ It may speed up the recovery of partial obstruction stimulating peristalsis and reducing wall oedema though there are insufficient evidence to determine its position in the prediction of palliative patients that may respond to such therapy.^10,22–25^ A meta-analysis suggests that the true value of diatrizoate meglumine lies in its ability to determine the timing of surgery in patients with adhesive small bowel obstruction.^26^ Notably, no significant complications have been reported with the use of diatrizoate meglumine; however, general safety precautions applicable to contrast agents should still be observed.^10,27^ In nonresponders, pharmacological management with a combination of analgesics, antiemetics and antisecretory drugs improves gastrointestinal symptoms (Table 1).^19,28,29^

**TABLE 1.** Summary of the pharmacological treatment frequently used in malignant bowel obstruction

Substance class	Drug	Remark
Analgesic	morphine	For pain – titrate or according to previous dosage
Corticosteroids	dexamethasone*	Peri-tumourous oedema reduction and anti-emetic effect
Somatostatine analagues	octreotide*	Reduction of gastrintestinal secrections
Anti-cholinergic	buthylscopolamine*	Reduction of gastrintestinal secrections
Prokinetic agent	metoclopramide	Drug of choice for incomplete obstruction; due to increased gastrointestinal motility, pain and vomiting may worsen; CAVE – for complete obstruction
Anti-psychotic	haloperidol*	Drug of choice for complete obstruction
	levomepromazine*	
	olanzapine*	CAVE – elderly, patients with demetia
Setrone	granisetron*	Increases constipation
H_2_ blocker	ranitidine*	
Proton pump inhibitor	omeprazole*	

* = off-label use

Causes of pain in MBO are abdominal distention and colic.^10^ Due to the lack of studies specifically addressing the use of opioids for pain management in MBO, the supporting evidence is limited; nevertheless, opioids are commonly used in clinical practice for this purpose.^10,30^ They act directly on pain due to occlusion but also reduce bowel contractions against the obstruction site(s).^31^ Preferably analgesics should be given by mouth, but due to vomiting and nausea, parenteral, subcutaneous and/or transdermal routes should be considered especially in complete MBO.^10,30^ Patients with MBO may experience malignant visceral pain due to organ obstruction and mesenteric infiltration.^32^ In literature, lidocaine infusion was proposed as an effective and safe alternative for visceral pain in patients with MBO.^32,34^ Moreover, lidocaine infusion accelerates bowel recovery after surgery and improves defecation.^35,36^ Corticosteroids could be beneficial in the management of pain, nausea and vomiting in MBO probably due to their anti-inflammatory and anti-secretory effect.^10,37^ There are inconclusive data regarding the optimal dose.^38,39^ Spasmodic pain/colic following gastrointestinal secretions and propulsive peristalsis could be controlled by anticholinergics. Despite insufficient data supporting greater effectiveness of somatostatin analogue octreotide compared to the anticholinergic hyoscine butylbromide, large and long experience with octreotide suggests that octreotide is the drug of choice in MBO, especially to control vomiting.^3,10,40–48^ The mechanism that octreotide increases water absorption and improves bowel transition has not been experimentally validated yet.^49^ Potential inferiority of hyoscine butylbromide could be due to the necessity of requiring high therapeutic doses for efficacy.^3,48^ Studies suggested haloperidol use as anti-emetic in the complete MBO.^30,50^ Dopamine antagonist prokinetic drugs (metoclopramide) were found effective for relieving nausea and vomiting but also to restore bowel transit in the partial MBO.^10,28^ In a small study of patients with MBO due to advanced cancer, granisetron combined with dexamethasone was effective in controlling emesis that was refractory to other antiemetics, although dexamethasone alone may also exert antiemetic effects.^51^ Histamine antagonists (cyclizine), phenothiazines, serotonin antagonists and thienobenzodiazepine antipsychotics may reduce nausea and vomiting, but there are no randomized trials to evidence their effect.^10,50^ Based on a meta-analysis, research should be directed to the role of histamine-2 antagonists as adjuvant antisecretory agents in MBO as they have been found to be superior to proton pump inhibitors in reducing gastric secretions (Table 1).^52^ A combination of different agents can act synergistically.^3,10,28^ A continuous subcutaneous infusion with combinations of different agents is a reliable and feasible modality to apply pharmacological treatment in different settings.^30,53^

Table 2 provides a summary of suggestions and recommendations for the pharmacological management of pain and gastrointestinal symptoms in patients with MBO and advanced cancer with a combination of analgesics, antiemetics and antise-cretory drugs in accordance to MASCC suggesstions.^10^

**TABLE 2.** Summary of suggestions and recommendations for malignant bowel obstruction management, with associated level and grade of evidence^10^

Intervention	Level of evidence^1^	Grade^2^
***Anti-emetics***		
Anticholinergics (e.g., hyoscine butylbromide) are generally less effective than octreotide for reducing vomiting in MBO.	III	D
Haloperidol demonstrates anti-emetic efficacy, particularly in complete MBO.	IV	B
Dopamine antagonist prokinetic drugs (metoclopramide) may be beneficial in partial MBO but is generally contraindicated in complete MBO due to the risk of perforation.	III	B
Histamine H1 antagonists, (e.g., dimenhydrinate, cyclizine) show utility in nausea and vomiting reduction in complete MBO.	IV	D
Phenothiazines (e.g., chlorpromazine) may be usful anti-emetics in MBO.	IV	D
Granisetron, serotonin (5HT3) antagonist may decrease nausea and vomiting in MBO.	III	D
Somatostatin analog (octreotide, lanreotide) may decrease vomiting in MBO.	I	A
Thienobenzodiazepene antipsychotic (e.g., olanzapine) may provide benefit in reducing nausea and vomiting in MBO.	I	A
***Analgesics***		
Although there is no evidence of support, opioids are commonly used to treat pain associated with MBO.	V	D
Anticholinergics (hyoscine butylbromide) may be effective in abdominal pain reduction in MBO.	III	D
***Corticosteroids***		
Steroids may reduce acute symptoms of MBO and be used for short-term benefits.	III	B
***Bowel decompression***		
Nasogastric tube may be temporary used for decompression in acute MBO.	V	D
Endoscopic or percutaneous gastrostomy tube may help in gastric decompression in MBO.	IV	B
Percutaneous transesophageal gastro-tubing may be help in gastric decompression in MBO.	IV	C

1 Level of evidence (Table 3);

2 Grade (Table 4); MBO = malignant bowel obstruction

**TABLE 3.** Level of evidence.^10^ Level I and II are reccomendations, III-V suggestions

Level	Criteria
I	Meta-analysis of multiple, well-designed, controlled studies; randomized trials with high power.
II	At least one-well designed experimental study; randomized trials with low power.
III	Well-designed, quasi-experimental studies (nonrandomized, controlled single-group, pretest-posttest comparison, cohort, time, or matched case-control series).
IV	Well-designed, non-experimental studies (comparative and correlational descriptive and case studies).
V	Case reports and clinical examples.

**TABLE 4.** Grade^10^

Grade	Evidence needed
A	Type I or consistent findings from multiple studies of type II, III, or IV.
B	Types II, III, or IV and consistent findings.
C	Types II, III, or IV and inconsistent findings.
D	Little/no systematic empirical evidence.

Nasogastric tube (NGT) is an established measure used as a venting/decompression procedure in MBO.^10^ It is frequently used to evacuate pooled gastric secretions in acute episodes of MBO.^22^ Decompression due to NGT placement reduces the risk for aspiration, which is associated to high mortality rate in case of vomiting.^10^ However, longterm NGT is poorly tolerated because of occlusion, displacement and intrusive appearance and causes complications (nasal cartilage erosion, bleeding, sinusitis, aspiration pneumonia).^10,22^ As a part of an aggressive conservative management strategy in MBO, long intestinal tubes (LITs) could be complementary to NGT.^54^ LIT enables suction closer to the obstruction and could be more efficient in providing intraluminal decompression in case of small bowel obstruction.^54^ A very small study from Canada described the positioning of a percutaneous tranesophageal gastrostomy (PTEG) as an effective solution for gastric drainage.^15^ Only minor complications were described with half of the patients (5/10) being discharged from the hospital in a week.^15^

Endoscopic gastric decompression is advocated due to frequent complications and a high mortality level associated with palliative surgical intervention.^5^ Decompressive percutaneous endoscopic gastrostomy (PEG) tubes represent a safe and an effective alternative in the management of MBO symptoms, sometimes allowing patients some oral intake (liquids, soft foods) and thus improving the social aspect of the QoL of patients.^55,56^ Minor contraindications to PEG are ascites and carcinomatosis. In case patients are unfit for palliative surgery and have an estimated life expectancy of more than 30 days, pharmacological treatment and NGT should be considered first.^55^ PEG is indicated when drugs fail to reduce vomiting in case patient’s performance status (and prognosis) are permitting such a procedure.^2,55,57^ Interventional radiology guided gastrostomy tubes insertion may be an even less invasive procedure.^58^

Stents (self-expandable metallic stent (SEMS)) insertion while a less durable procedure compared to surgery is also a feasible and safe therapeutic possibility for palliation or for bridging to chemotherapy or surgery even in elderly patients and an alternative to palliative surgery.^13,14,59,60^ Endoscopic stent may obviate the need for an intestinal stoma.^61^ Stenting also includes procedures intent to canalize the lumen.^31^ Endoluminal wall stents have a high success rate for improvement of symptoms caused of MBO in case of complete and incomplete obstruction as well as in upper intestinal obstructions (gastric outlet, duodenal and jejunal obstructions). Although risk include perforation, stent migration and reocclusion, stents may be a definitive palliative treatment and may offer adequate palliation for longer periods (months).^31,62,63^ Stents are effective for primary left-sided obstructions.^64^ Studies suggested that they may also be applicable in cases of lower rectal obstruction, which had previously been considered a contraindicated area for stent placement.^62,65,66^ The results of a retrospective single centre study that observed data from SEMS insertion between 2014 and 2020 highlighted that SEMS are a good alternative compared to palliative surgery in respect to high perioperative morbidity.^59^ This somehow contrasts a small single institution study where patients undergoing palliative interventions had a shortest survival compared to patients managed with palliative surgery and conservative/pharmacological treatment what could be related to a more advanced disease in those patients with worse prognosis who were deemed inoperable.^12^

Table 5 provides a summary of recommendations for palliative (non-surgical) management of pain and gastrointestinal symptoms in patients with MBO and advanced cancer in accordance to MASCC suggesstions.^10^

**TABLE 5.** Summary of recommendations for malignant bowel obstruction management, with associated level and grade of evidence.^10^ Level of evidence (Table 3), Grade (Table 4)

Palliative (non-surgical) procedures	Level of evidence^1^	Grade^2^
Self-expanding metallic stents represent the preferred option for managing single-level large bowel obstruction, provided the procedure is technically achievable and there is no evidence of colonic perforation.	II	B

1 Level of evidence (Table 3);

2 Grade (Table 4)

Persistent obstructions despite comfort-focused care (conservative/pharmacological treatment) – typically including pharmacological treatment, nasogastric decompression and bowel rest – or evidence of complete obstruction may indicate the need for palliative surgical intervention, but many times (6.2–50%) patients are not suitable for palliative surgery.^31,67^ MBO is one of the leading causes for surgical consultations in patients with advanced cancer.^68^ Palliative surgery is routinelynot advisable in patients with poor prognostic criteria.^2,7^ Clear criteria for the selection of patients eligible for palliative surgery are still needed.^69^ Scores on which selection is based are age, comorbidities, nutritional status, tumour status (palpable masses), multiple MBOs, carcinomatosis, presence of ascites, previous specific oncological therapy (chemotherapy, radiotherapy) and short time from treatment to obstruction. In addition to previously mentioned characteristic studies suggest considering performance status of the patient.^7,70^ Potential contraindications for palliative surgery in patients with advanced cancer and MBO are ascites, carcinomatosis, particularly the combination of ascites and carcinomatosis, multiple MBOs, low albumin, prior surgeries and palpable intraabdominal masses.^11^ Laparoscopic procedures may be attempted but due to adhesions, carcinomatosis and bowel dilatations are often difficult to perform.^31^ Surgical interventions are related to complications and risk for hospital (re)admissions as well as high postoperative mortality (9–40%) and morbidity (9–90%) although in a small single institution study surgical patient had despite longer initial hospital stays and higher resource use lower readmission rates and better QoL.^5,12,71^ Improvement in QoL after palliative surgery for MBO has been reported to vary widely (42–85%) but tends to be greater in carefully selected patients considered fit for palliative surgery, compared with those managed using pharmacological or non-surgical approaches.^12,31,67,71,72^ Patients who underwent palliative surgery have better oral food intake.^71^ Palliative surgery (resection, bypass, diversion), compared to venting gastrostomy tubes that provide drainage and symptom relief, is meant to treat the underlying disease.^9^ In some cases, obstructions may spontaneously resolve, but recurrent episodes are common with high rates of hospital admissions and poor QoL in patients at the EoL.^9^ Palliative surgery can potentially allow patients to resume specific oncological treatments, which is rarely possible for patients at the EoL.^73^ Studies directed in the factors predicting patients to eventually resume oncological treatment would allow clinicians easier selection of patients where more aggressive treatment to resolve MBO could potentially be beneficial. Table 6 provides a summary of suggestions for palliative surgery in patients with MBO and advanced cancer in accordance to MASCC suggesstions.^10^

**TABLE 6.** Summary of suggestions for palliative surgery for malignant bowel obstruction management, with associated level and grade of evidence.^10^ Level of evidence (Table 3), Grade (Table 4)

Palliative surgery	Level of evidence^1^	Grade^1^
For patients with multi-level obstruction, palliative surgical intervention may be appropriate in carefully selected cases.	IV	B
Patients with advanced cancer who undergo palliative surgery for MBO face a substantial risk of postoperative complications; therefore, less invasive surgical approaches should be considered whenever feasible.	IV	B

1 Level of evidence (Table 3);

1 ^2^ Grade (Table 4); MBO = malignant bowel obstruction

Oral intake could be significantly impaired, especially in the complete MBO. Starting with nil-by-mouth and slowly, when possible – as symptoms resolve, reintroducing an oral diet with clear to full fluids and when amendable a low fibre diet is recommended and a part of non-pharmacological interventions suggested in the literature.^1^ Nutritional interventions – nutritional assessment and introduction of total parenteral nutrition – are controversial and ethically challenging.^74^ Most patients with advanced cancer disease may not benefit from total parenteral nutrition.^75^ Additional studies would be beneficial for a better selection of patients suitable for this kind of interventions.

It is important to balance well between the efficacy of hydration (daily thirst, dry mouth, nausea) and the additional production of bowel secretions.^30,76^ A controlled randomized study involving patients at the end of life showed that a subcutaneous infusion of 1000 ml saline did not improve patients’ symptoms nor their quantity or QoL.^77^

Table 7 provides a summary of suggestions for nutrition in patients with MBO and advanced cancer in accordance to MASCC suggesstions.^10^

**TABLE 7.** Summary of suggestions for malignant bowel obstruction management, with associated level and grade of evidence^10^

Nutrition	Level of evidence^1^	Grade^2^
At the initial diagnosis of MBO, patients should be placed nil per os. Once the obstruction has fully or partially resolved, a gradual, symptom-guided reintroduction of oral intake is recommended. This typically progresses from clear fluids to free or full fluids, followed by texture-modified low-fiber diets, and, if tolerated, a return to a normal-textured low-fiber diet.	IV	B
Nutrition interventions for patients with advanced cancer should be pursued only when the anticipated benefits for quality of life or survival clearly outweigh the associated risks. These decisions should be guided by a multidisciplinary team and include explicit discussions with patients and their caregivers about expected outcomes.	IV	B
Parenteral hydration has not been shown to prevent or alleviate symptoms such as thirst or dry mouth, nor does it prolong survival. When administered in excess, it may contribute to fluid overload and the development of peripheral or pulmonary edema.	III	B
Routine initiation of parenteral hydration is not recommended during the last days of life.	III	B
Home parenteral nutrition may provide benefit and help preserve quality of life in a carefully selected subset of patients with MBO.	IV	D
For home parenteral nutrition delivery central venous access is preferred.	III	B
At the end-of-life at home, parenteral nutrition should be discontinued (or not initiated) as it raises the risk of complications and may prolong suffering.	V	D

1 Level of evidence (Table 3),

2 Grade (Table 4); MBO = malignant bowel obstruction

Even in patients referred to palliative care psychosocial needs are frequently unmet and poorly addressed.^78,79^ Reference to early palliative care could improve patients’ QoL also in terms of a psychosocial support as preparedness for disease progression over time.^80,81^

Goals of treatment should be individualized in relation to patients’ priorities and feasible options. Many patients express a preference for discharge, wishing to spend the end of life at home in the presence of family and friends.^82,83^

### Limitations

This review has several limitations. First, only one database was searched, which may have restricted the breadth and completeness of the retrieved literature. Furthermore, no formal quality assessment of individual studies were conducted, limiting the ability to evaluate the robustness and potential bias of the included evidence. For these reasons, the review does not meet the criteria of a systematic review in the strict methodological sense. In addition, the search strategy was intentionally broad, which required substantial manual filtering to identify studies specifically relevant to advanced cancer and EoL populations. This approach may have introduced selection subjectivity and the possibility of overlooking pertinent studies.

## Conclusions

The best symptoms’ management in patients at the EoL and MBO is problematic as there is no gold standard and it varies related to patients’ clinical condition and status of advanced disease. Nonetheless, clinical trials to assess the QoL of patients with very advanced cancer are difficult to conduct and many times ethically challenging. Thus symptom managing in MBO should be a multidisciplinary approach including comfort-focused care (conservative/pharmacological treatments), palliative (non-surgical) procedures and palliative surgery with focus on minimizing symptoms and maximizing the QoL.

Patients undergoing palliative surgery have generally better prognostic criteria and higher level of physical conditioning compared to patients deemed inoperable. Palliative surgery is related to high perioperative mortality and morbidity, but studies report longer and better QoL in a highly selected patients’ population with increase in life expectancy. Patients treated with comfort-focused (conservative/pharmacological treatment) tend to have higher morbidity at MBO presentation and compared to surgical and non-surgical (procedural) manged patients’ shorter survival times with less days in hospital and in-hospital deaths.

It is essential to engage patients and their families in goals-of-care discussions and advanced care planning to promote understanding of the palliative care intent of MBO interventions, carefully and individually weigh the risks and benefits of all treatment options, and collaboratively determine the most appropriate approach to symptom management.

The goals of treatment in MBO and advanced cancer are usually symptoms control – pain, nausea and vomiting – and the allowance of some oral intake – usually clear to full liquids and sometimes a liquid low fibre diet. Goals of treatment should be individualized in relation to patients’ priorities and feasible options.

The introduction of an early palliative care for patients with an advanced disease could allow a thoughtful and resourceful care and discussions that could benefit patients throughout their disease.

## References

[1] Yu K, Liu L, Zhang X, Zhang Z, Rao B, Chen Y (2020). Surgical and conservative management of malignant bowel obstruction: outcome and prognostic factors. Cancer Manag Res.

[2] Ripamonti C, Twycross R, Baines M, Bozzetti F, Capri S, De Conno F (2001). Clinical-practice recommendations for the management of bowel obstruction in patients with end-stage cancer. Support Care Cancer.

[3] Davis M, Hui D, Davies A, Ripamonti C, Capela A, DeFeo G (2021). Medical management of malignant bowel obstruction in patients with advanced cancer: 2021 MASCC guideline update. Support Care Cancer.

[4] Franke AJ, Iqbal A, Starr JS, Nair RM, George TJ. (2017). Management of malignant bowel obstruction associated with GI cancers. J Oncol Pract.

[5] Cole JM, Khan SZ, Marks JM, Armstrong AJ, Zanotti KM, Juza RM. (2023). Enhanced gastric decompression for palliation of malignant bowel obstruction. Surg Endosc.

[6] Pameijer CR, Mahvi DM, Stewart JA, Weber SM. (2005). Bowel obstruction in patients with metastatic cancer: does intervention influence outcome?. Int J Gastrointest Cancer.

[7] Daniele A, Ferrero A, Fuso L, Mineccia M, Porcellana V, Vassallo D (2015). Palliative care in patients with ovarian cancer and bowel obstruction. Support Care Cancer.

[8] Tuca A, Guell E, Martinez-Losada E, Codorniu N. (2012). Malignant bowel obstruction in advanced cancer patients: epidemiology, management, and factors influencing spontaneous resolution. Cancer Manag Res.

[9] Sun BJ, Tennakoon L, Spain DA, Lee B. (2024). Palliative intervention for malignant bowel obstruction comes at a cost: a national inpatient study. Am Surg.

[10] Madariaga A, Lau J, Ghoshal A, Dzierżanowski T, Larkin P, Sobocki J (2022). MASCC multidisciplinary evidence-based recommendations for the management of malignant bowel obstruction in advanced cancer. Support Care Cancer.

[11] Krouse RS. (2007). The international conference on malignant bowel obstruction: a meeting of the minds to advance palliative care research. J Pain Symptom Manage.

[12] Sun BJ, Yue TM, Xu N, Ayala CI, Lee B. (2024). Surgical palliation for malignant bowel obstruction in preventing hospital readmission: experience of a tertiary care center. Ann Surg Oncol.

[13] Horesh N, Dux JY, Nadler M, Lang A, Zmora O, Shacham-Shmueli E (2016). Stenting in malignant colonic obstruction – is it a real therapeutic option?. Int J Colorectal Dis.

[14] Imai M, Kamimura K, Takahashi Y, Sato T, Isokawa O, Maruyama M (2018). The factors influencing long-term outcomes of stenting for malignant colorectal obstruction in elderly group in community medicine. Int J Colorectal Dis.

[15] Selby D, Nolen A, Sittambalam C, Johansen K, Pugash R. (2019). Percutaneous transesophageal gastrostomy (PTEG): a safe and well-tolerated procedure for palliation of end-stage malignant bowel obstruction. J Pain Symptom Manage.

[16] Borowiec AM, Wang CSK, Yong E, Law C, Coburn N, Sutradhar R (2016). Colonic stents for colorectal cancer are seldom used and mainly for palliation of obstruction: a population-based study. Can J Gastroenterol Hepatol.

[17] Ahn HJ, Kim SW, Lee SW, Lee SW, Lim CH, Kim JS (2016). Long-term outcomes of palliation for unresectable colorectal cancer obstruction in patients with good performance status: endoscopic stent versus surgery. Surg Endosc.

[18] Rademacher C, Bechtler M, Schneider S, Hartmann B, Striegel J, Jakobs R. (2016). Self-expanding metal stents for the palliation of malignant gastric outlet obstruction in patients with peritoneal carcinomatosis. World J Gastroenterol.

[19] Tóth R, Tóth Z, Lőczi L, Török M, Ács N, Várbíró S (2024). Management of malignant bowel obstruction in patients with gynaecological cancer: a systematic review. J Clin Med.

[20] Neri B, Citterio N, Schiavone SC, Biasutto D, Rea R, Martino M (2025). Malignant bowel occlusion: an update on current available treatments. Cancers (Basel).

[21] Hisanaga T, Shinjo T, Imai K, Katayama K, Kaneishi K, Honma H (2019). Clinical guidelines for management of gastrointestinal symptoms in cancer patients: the Japanese Society of Palliative Medicine recommendations. J Palliat Med.

[22] Tutino R, Cavaglià M, Pipitone Federico NS, De Simone V, Deiro G, Gallo G (2025). The diagnostic and therapeutic value of Gastrografin in small bowel obstructions. Front Surg.

[23] Wan Bahrum WFI Bin, Hardy J, Foster K, Good P. (2023). Oral water-soluble contrast for malignant bowel obstruction: open label pilot study. BMJ Support Palliat Care.

[24] Syrmis W, Richard R, Jenkins-Marsh S, Chia SC, Good P. (2018). Oral water soluble contrast for malignant bowel obstruction. Cochrane Database Syst Rev.

[25] Zhang Y, Gao Y, Ma Q, Dang C, Wei W, Deantoni F (2006). Randomised clinical trial investigating the effects of combined administration of octreotide and methylglucamine diatrizoate in older persons with adhesive small bowel obstruction. Dig Liver Dis.

[26] Branco BC, Barmparas G, Schnüriger B, Inaba K, Chan LS, Demetriades D. (2010). Systematic review and meta-analysis of the diagnostic and therapeutic role of water-soluble contrast agent in adhesive small bowel obstruction. Br J Surg.

[27] Heng S, Hardy J, Good P. (2018). A retrospective audit on usage of diatrizoate meglumine (Gastrografin) for intestinal obstruction or constipation in patients with advanced neoplasms. Palliat Med.

[28] Mercadante S, Ferrera P, Villari P, Marrazzo A. (2004). Aggressive pharmacological treatment for reversing malignant bowel obstruction. J Pain Symptom Manage.

[29] Walter M, Hansen E, Hamid S, Carozza D, Mann G, Roche C (2024). Palliative management of inoperable malignant bowel obstruction: prospective, open label, phase 2 study at an NCI comprehensive cancer center. J Pain Symptom Manage.

[30] Ventafridda V, Ripamonti C, Caraceni A, Spoldi E, Messina L, De Conno F. (1990). The management of inoperable gastrointestinal obstruction in terminal cancer patients. Tumori.

[31] Krouse RS. (2019). Malignant bowel obstruction. J Surg Oncol.

[32] Velilla Echeverri DC, Gómez Díaz M, Beltrán Pachón P, Lasso Valenzuela D, Poveda Carreño S, Erazo-Muñoz M. (2023). Lidocaine infusion for malignant visceral pain: case report. BMJ Support Palliat Care.

[33] Mudumbi SK, Leonard EV, Swetz KM. (2017). Challenges and successes in nonoperative management of high-grade malignant bowel obstruction. Ann Palliat Med.

[34] Swenson BR, Gottschalk A, Wells LT, Rowlingson JC, Thompson PW, Barclay M (2010). Intravenous lidocaine is as effective as epidural bupivacaine in reducing ileus duration, hospital stay, and pain after open colon resection. Reg Anesth Pain Med.

[35] Chen PC, Lai CH, Fang CJ, Lai PC, Huang YT. (2022). Intravenous infusion of lidocaine for bowel function recovery after major colorectal surgery: a critical appraisal through updated meta-analysis, trial sequential analysis, certainty of evidence, and meta-regression. Front Med (Lausanne).

[36] Harvey KP, Adair JD, Isho M, Robinson R. (2009). Can intravenous lidocaine decrease postsurgical ileus and shorten hospital stay in elective bowel surgery? A pilot study and literature review. Am J Surg.

[37] Philip J, Lickiss N, Grant PT, Hacker NF. (1999). Corticosteroids in the management of bowel obstruction on a gynecological oncology unit. Gynecol Oncol.

[38] Feuer DJ, Broadley KE. (2000). Corticosteroids for the resolution of malignant bowel obstruction in advanced gynaecological and gastrointestinal cancer. Cochrane Database Syst Rev.

[39] Laval G, Girardier J, Lassaunière JM, Leduc B, Haond C, Schaerer R. (2000). The use of steroids in the management of inoperable intestinal obstruction in terminal cancer patients: do they remove the obstruction?. Palliat Med.

[40] Mercadante S, Porzio G. (2012). Octreotide for malignant bowel obstruction: Twenty years after. Crit Rev Oncol Hematol.

[41] Khoo D, Motson R, Denman K, Hall E, Riley J, Waxman J. (1994). Palliation of malignant intestinal obstruction using octreotide. Eur J Cancer.

[42] Kubota H, Taguchi K, Kobayashi D, Naruyama H, Hirose M, Fukuta K (2013). Clinical impact of palliative treatment using octreotide for inoperable malignant bowel obstruction caused by advanced urological cancer. Asian Pac J Cancer Prev.

[43] Laval G, Rousselot H, Toussaint-Martel S, Mayer F, Terrebonne É, François É (2012). SALTO: a randomized, multicenter study assessing octreotide LAR in inoperable bowel obstruction. Bull Cancer.

[44] Hisanaga T, Shinjo T, Morita T, Nakajima N, Ikenaga M, Tanimizu M (2010). Multicenter prospective study on efficacy and safety of octreotide for inoperable malignant bowel obstruction. Jpn J Clin Oncol.

[45] Shima Y, Ohtsu A, Shirao K, Sasaki Y. (2008). Clinical efficacy and safety of octreotide (SMS201-995) in terminally ill Japanese cancer patients with malignant bowel obstruction. Jpn J Clin Oncol.

[46] Massacesi C, Galeazzi G. (2006). Sustained release octreotide may have a role in the treatment of malignant bowel obstruction. Palliat Med.

[47] Mercadante S, Spoldi E, Caraceni A, Maddaloni S, Simonetti MT. (1993). Octreotide in relieving gastrointestinal symptoms due to bowel obstruction. Palliat Med.

[48] Peng X, Wang P, Li S, Zhang G, Hu S. (2015). Randomized clinical trial comparing octreotide and scopolamine butylbromide in symptom control of patients with inoperable bowel obstruction due to advanced ovarian cancer. World J Surg Oncol.

[49] Shinjo T, Kagami R. (2009). Radiological imaging change in a malignant bowel obstruction patient treated with octreotide. Support Care Cancer.

[50] Fainsinger RL, Spachynski K, Hanson J, Bruera E. (1994). Symptom control in terminally ill patients with malignant bowel obstruction (MBO). J Pain Symptom Manage.

[51] Tuca A, Roca R, Sala C, Porta J, Serrano G, González-Barboteo J (2009). Efficacy of granisetron in the antiemetic control of nonsurgical intestinal obstruction in advanced cancer: a phase II clinical trial. J Pain Symptom Manage.

[52] Clark K, Lam L, Currow D. (2009). Reducing gastric secretions – a role for histamine 2 antagonists or proton pump inhibitors in malignant bowel obstruction?. Support Care Cancer.

[53] Thompson I. (2004). The management of nausea and vomiting in palliative care. Nurs Stand.

[54] Jeong WK, Lim SB, Choi HS, Jeong SY. (2008). Conservative management of adhesive small bowel obstructions in patients previously operated on for primary colorectal cancer. J Gastrointest Surg.

[55] Zucchi E, Fornasarig M, Martella L, Maiero S, Lucia E, Borsatti E (2016). Decompressive percutaneous endoscopic gastrostomy in advanced cancer patients with small-bowel obstruction is feasible and effective: a large prospective study. Support Care Cancer.

[56] Campagnutta E, Cannizzaro R, Gallo A, Zarrelli A, Valentini M, De Cicco M (1996). Palliative treatment of upper intestinal obstruction by gynecological malignancy: the usefulness of percutaneous endoscopic gastrostomy. Gynecol Oncol.

[57] Brooksbank MA, Game PA, Ashby MA. (2002). Palliative venting gastrostomy in malignant intestinal obstruction. Palliat Med.

[58] Gauvin G, Do-Nguyen CC, Lou J, O‘Halloran EA, Selesner LT, Handorf E (2021). Gastrostomy tube for nutrition and malignant bowel obstruction in patients with cancer. J Natl Compr Canc Netw.

[59] Papachrysos N, Shafazand M, Alkelin L, Kilincalp S, de Lange T. (2023). Outcome of self-expandable metal stents placement for obstructive colorectal cancer: 7 years’ experience from a Swedish tertiary center. Surg Endosc.

[60] Khot UP, Lang AW, Murali K, Parker MC. (2002). Systematic review of the efficacy and safety of colorectal stents. Br J Surg.

[61] Hünerbein M, Krause M, Moesta KT, Rau B, Schlag PM. (2005). Palliation of malignant rectal obstruction with self-expanding metal stents. Surgery.

[62] Spinelli P, Mancini A. (2001). Use of self-expanding metal stents for palliation of rectosigmoid cancer. Gastrointest Endosc.

[63] de Gregorio MA, Mainar A, Tejero E, Tobío R, Alfonso E, Pinto I (1998). Acute colorectal obstruction: stent placement for palliative treatment – results of a multicenter study. Radiology.

[64] van Hooft J, van Halsema E, Vanbiervliet G, Beets-Tan R, DeWitt J, Donnellan F (2014). Self-expandable metal stents for obstructing colonic and extracolonic cancer: ESGE Clinical Guideline. Endoscopy.

[65] Lee KM, Lim SG, Shin SJ, Kim JH, Kang DH, Kim JK (2013). Novel method of stent insertion for malignant lower rectal obstruction with proximal releasing delivery system. Gastrointest Endosc.

[66] Friedland S, Hallenbeck J, Soetikno RM. (2001). Stenting the sigmoid colon in a terminally ill patient with prostate cancer. J Palliat Med.

[67] Feuer DJ, Broadley KE, Shepherd JH, Barton DPJ. (1999). Systematic review of surgery in malignant bowel obstruction in advanced gynecological and gastrointestinal cancer. Gynecol Oncol.

[68] Badgwell BD, Smith K, Liu P, Bruera E, Curley SA, Cormier JN. (2009). Indicators of surgery and survival in oncology inpatients requiring surgical evaluation for palliation. Support Care Cancer.

[69] Zielinski MD, Eiken PW, Heller SF, Lohse CM, Huebner M, Sarr MG (2011). Prospective, observational validation of a multivariate small-bowel obstruction model to predict the need for operative intervention. J Am Coll Surg.

[70] Santangelo ML, Grifasi C, Criscitiello C, Giuliano M, Calogero A, Dodaro C (2017). Bowel obstruction and peritoneal carcinomatosis in the elderly: a systematic review. Aging Clin Exp Res.

[71] Akbaş A. (2023). Does a selective surgical approach to malignant bowel obstruction help in palliative care patients?. Turk J Trauma Emerg Surg.

[72] McCaffrey N, Asser T, Fazekas B, Muircroft W, Agar M, Clark K (2020). Healthrelated quality of life in patients with inoperable malignant bowel obstruction: secondary outcome from a double-blind, parallel, placebo-controlled randomised trial of octreotide. BMC Cancer.

[73] Helyer LK, Law CHL, Butler M, Last LD, Smith AJ, Wright FC. (2007). Surgery as a bridge to palliative chemotherapy in patients with malignant bowel obstruction from colorectal cancer. Ann Surg Oncol.

[74] Druml C, Ballmer PE, Druml W, Oehmichen F, Shenkin A, Singer P (2016). ESPEN guideline on ethical aspects of artificial nutrition and hydration. Clin Nutr.

[75] Patel PS, Fragkos K, Keane N, Wilkinson D, Johnson A, Chan D (2024). Nutritional care pathways in cancer patients with malignant bowel obstruction: a retrospective multi-centre study. Clin Nutr ESPEN.

[76] Ripamonti C, Mercadante S, Groff L, Zecca E, De Conno F, Casuccio A. (2000). Role of octreotide, scopolamine butylbromide, and hydration in symptom control of patients with inoperable bowel obstruction and nasogastric tubes. J Pain Symptom Manage.

[77] Bruera E, Hui D, Dalal S, Torres-Vigil I, Trumble J, Roosth J (2013). Parenteral hydration in patients with advanced cancer: a multicenter, double-blind, placebo-controlled randomized trial. J Clin Oncol.

[78] Hoppenot C, Hlubocky FJ, Chor J, Yamada SD, Lee NK. (2020). Approach to palliative care consultation for patients with malignant bowel obstruction in gynecologic oncology: a qualitative analysis of physician perspectives. JCO Oncol Pract.

[79] Selby D, Wright F, Stilos K, Daines P, Moravan V, Gill A (2010). Room for improvement? A quality-of-life assessment in patients with malignant bowel obstruction. Palliat Med.

[80] Temel JS, Greer JA, Muzikansky A, Gallagher ER, Admane S, Jackson VA (2010). Early palliative care for patients with metastatic non-small-cell lung cancer. N Engl J Med.

[81] Aggarwal G, Glare P, Clarke S, Chapuis P. (2006). Palliative and shared care concepts in patients with advanced colorectal cancer. ANZ J Surg.

[82] Kern H, Corani G, Huber D, Vermes N, Zaffalon M, Varini M (2020). Impact on place of death in cancer patients: a causal exploration in southern Switzerland. BMC Palliat Care.

[83] Gomes B, Higginson IJ, Calanzani N, Cohen J, Deliens L, Daveson BA (2012). Preferences for place of death if faced with advanced cancer: a population survey in seven European countries. Ann Oncol.

